# Self-assembly and condensation of intermolecular poly(UG) RNA quadruplexes

**DOI:** 10.1093/nar/gkae870

**Published:** 2024-10-07

**Authors:** Saeed Roschdi, Eric J Montemayor, Rahul Vivek, Craig A Bingman, Samuel E Butcher

**Affiliations:** Department of Biochemistry, University of Wisconsin-Madison, Madison, WI 53706, USA; Department of Biochemistry, University of Wisconsin-Madison, Madison, WI 53706, USA; Department of Biochemistry, University of Wisconsin-Madison, Madison, WI 53706, USA; Department of Biochemistry, University of Wisconsin-Madison, Madison, WI 53706, USA; Department of Biochemistry, University of Wisconsin-Madison, Madison, WI 53706, USA

## Abstract

Poly(UG) or ‘pUG’ dinucleotide repeats are highly abundant sequences in eukaryotic RNAs. In *Caenorhabditis elegans*, pUGs are added to RNA 3′ ends to direct gene silencing within *Mutator* foci, a germ granule condensate. Here, we show that pUG RNAs efficiently self-assemble into gel condensates through quadruplex (G4) interactions. Short pUG sequences form right-handed intermolecular G4s (pUG G4s), while longer pUGs form left-handed intramolecular G4s (pUG folds). We determined a 1.05 Å crystal structure of an intermolecular pUG G4, which reveals an eight stranded G4 dimer involving 48 nucleotides, 7 different G and U quartet conformations, 7 coordinated potassium ions, 8 sodium ions and a buried water molecule. A comparison of the intermolecular pUG G4 and intramolecular pUG fold structures provides insights into the molecular basis for G4 handedness and illustrates how a simple dinucleotide repeat sequence can form complex structures with diverse topologies.

## Graphical Abstract

**Graphical Abstract:**
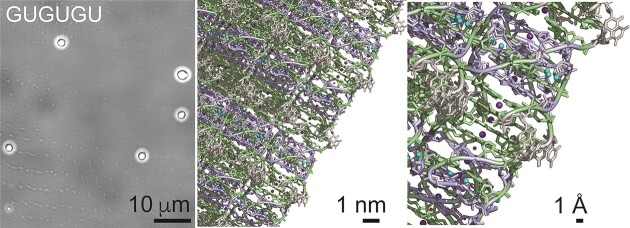

## Introduction

Germ granules are non-membrane bound compartments of condensed RNA and proteins that regulate gene expression during development of all metazoans (1–4). Germ granule condensates can be approximately a micron in diameter, equivalent to the size of bacteria (5), and have been most extensively studied in drosophila and *Caenorhabditis elegans* (1–5). However, many aspects of germ granule biology are not understood. In *C. elegans*, germ granules are multi-body condensates that include *Mutator* foci (2,6), which are sites of gene silencing and transgenerational epigenetic inheritance (TEI) (7). The formation of *Mutator* foci is a complex and spatiotemporally regulated process (6,8) and involves the phase separation of proteins (9). The RNAs within *Mutator* foci have been less well studied and may also contribute to the formation and structure of these condensates.

*Mutator* foci contain the enzyme RDE-3, which adds non-templated poly(UG) or poly(GU) tails (collectively, pUG tails) to mRNA 3′ ends (7). These pUG tails can be over 100 nucleotides long (7,10), and recruit the RNA dependent RNA polymerase (RdRP) RRF-1 to RNA for the synthesis of small interfering RNAs (siRNAs) (7,11,12). This gene silencing activity requires the pUG fold, an intramolecular left-handed parallel RNA quadruplex (G4) structure (11). The pUG fold requires 12 guanosines for formation of 3 G quartets (11). However, gene silencing activity *in vivo* becomes much more efficient with longer tails, with maximal activity observed at ∼18.5 repeats (11). Therefore, we wondered if pUG tails may have additional structure beyond the pUG fold. We also hypothesized that pUG RNA interactions may phase separate into condensates, as other guanosine-rich RNA repeat sequences have been observed to form condensates *in vitro* and in cells (13).

Poly(UG) dinucleotide repeats are extraordinarily abundant in eukaryotic RNA and their structural interactions are not completely understood. For example, human genes have ∼20 000 pUG sequences with ≥12 guanosines (11). The pUG fold formed by the sequence (GU)_12_ is the only known example of a left-handed RNA quadruplex structure (12) and cannot be predicted with current approaches such as AlphaFold3 (14). Thus, there is a general need to improve our understanding of RNA folding (15), even for sequences as simple as dinucleotide repeats.

Here, we show that pUG RNAs form G4 condensates *in vitro*. These condensates can be formed from short sequences that cannot adopt pUG folds. We observe that short pUGs with 3–5 repeats form intermolecular pUG G4s with right-handed helical topologies, opposite of the pUG fold. Depending on RNA concentration, pUGs with six repeats form either dimeric left-handed pUG folds or multimeric right-handed pUG G4s. We present the high-resolution structure of a right-handed intermolecular pUG G4, which reveals a multimeric G4 complex formed around a core of seven coordinated potassium ions. Comparison of the pUG fold (11) and pUG G4 structures reveals new insights into RNA folding, including the molecular basis for the handedness of G4 structures.

## Materials and methods

### RNA production

RNAs were synthesized either from Integrated DNA Technologies or Horizon Discovery. The synthesized RNAs were resuspended in water. RNA for fluorescence recovery after photobleaching (FRAP) studies contained a 5′ fluorescein (FAM) label. The sequences of all oligonucleotides used in the study are listed in Supplemental Table S1.

### RNA condensation

For phase separation experiments, RNA or FAM-labeled RNAs were 2.3 mg/ml in 20 mM bis–tris pH 7, 50 mM KCl, 10% polyethylene glycol 8000, heated to 90°C, and cooled to room temperature slowly. Samples were then diluted to 0.23 mg/ml in the same buffer and if needed 30 μM NMM was added before loading onto slides with tape spacers. RNA condensates were allowed to settle onto the glass surface for ∼10 min before imaging. The brightfield images were taken using a Nikon N Storm microscope system with an Apo TIRF 100× Oil objective. The maximum intensity z-projection of a single 4 μm optical slice was used for all images, made with Fiji (ImageJ). These data were recorded using Nikon software.

### Fluorescence recovery after photobleaching (FRAP)

RNA condensates were prepared as above and imaged using a Nikon A1R-Si+ Confocal Microscope with a Plan Apo λ 60× Oil. A roughly 1.5 μm^2^ region was bleached at 50% laser power with either a 440 or 404 nm laser for 10 s and the fluorescence recovery was monitored by time-lapse imaging for 75 s at 1 frame/s. For the FAM samples, a 440 nm laser was used for excitation and a 525 nm emission filter was used. For NMM a 404 nM laser was used for excitation with a virtual filter set to 600–620 nm was used for emission. The fluorescence intensity of the bleached region was quantified using Fiji (ImageJ), then normalized and corrected for photobleaching.

### Circular dichroism (CD) spectroscopy

CD RNA samples contained various RNA concentrations in 20 mM bis–tris buffer pH 7.0 and either 140 mM KCl, 10 mM NaCl, and 2 mM MgCl_2_ or 20 mM bis–tris buffer pH 7.0 and 150 mM LiCl and annealed by incubation in 1 l 90°C water and slowly cool to room temperature. CD spectra were recorded in an AVIV model 420 CD spectrometer using a quartz cell with an adjustable optical path length of either 0.1, 0.2 or 1 mm. To keep the signal within the linear range of the instrument, the high RNA concentration samples (1–1.5 mM) were measured with the shortest optical path length (0.1 mm) and the lowest RNA concentration samples were measured with the long optical path length (1 mm). Scans were carried out with a step size of 1 nm and 5-s averaging times, and measurements were taken from 210 to 340 nm. Spectra were measured at 25°C with buffer subtraction, and data were converted to molecular CD absorption (Δϵ)


\begin{equation*}\Delta \varepsilon = \frac{\theta }{{32980 \times C \times L \times N}}\end{equation*}

where θ is the raw CD signal (in millidegrees), *C* is the RNA concentration (in M), *L* is the cuvette path length (in cm) and *N* is the number of nucleotides. The concentration dependence profile was characterized by the millidegree signal at 250 and 290 nm to determine the hill coefficient (*n*) and the complete folding concentration by fitting the data to the Hill equation using Origin (Origin 2020 OriginLab corporation).

### Crystallization and structure determination

High-throughput crystallization screening was initially performed on a Mosquito crystallization robot (TTP Labtech). Diffraction quality crystals were eventually obtained by sitting drop vapor diffusion by mixing 2 μl of RNA at 7.29 mM (14.3 mg/ml) in 100 mM KCl and 20 mM bis–tris buffer pH 6.5) with 2 μl of crystallization reagent containing 200 mM NaCl, 100 mM bis–tris pH 6.5 and 50% PEG 550 MME. Crystals were vitrified by direct immersion into liquid nitrogen. A heavy atom derivative was prepared by adding 20 μl of crystallization reagent saturated with holmium (III) acetate to a drop with pre-grown crystals and allowing the mixture to incubate in a sealed crystallization plate overnight at 298 K. Heavy atom derivative crystals were directly frozen without any subsequent buffer exchange.

Diffraction data were recorded at APS beamline 21-ID-D and integrated using XDS (16). Space group determination was performed in *POINTLESS* (17). *Phenix.xtriage* was used to assay potential twinning in the diffraction data (18). A heavy atom derivative was obtained by soaking crystals in Ho^3+^. Both native and Ho^3+^ derivatized RNAs yielded the same crystal form. Experimental phases and an initial electron density map were calculated by the method of Single Isomorphous Replacement with Anomalous Scattering, using initial heavy atom site identification, map calculation and density modification in the *SHELXC/D/E* pipeline (19) as implemented in *HKL2Map* (20). Structure refinement was performed in *Phenix.refine* (18,21)

Data were processed with autoproc (22) using XDS (16), pointless (23), and aimless (24) for isotropic scaling. The high-resolution limit of included data was limited by completeness (63% complete in the 1.071–1.052 Å shell, *I*/sigma(*I*) = 2.14). Refinement was conducted in the Phenix suite (25) using alternating cycles of Phenix.refine (21) and manual rebuilding in Coot (26). It was apparent that there was substantial conformational variability in both the backbone and to a lesser extent, the nucleobase positions. Alternative refinements were pursued using data processed in the isomorphic subgroups *C*222, *P*222_1_, with a half-tetramer in the asymmetric unit, and *P*2_1_, with a complete tetramer and four crystallographically independent strands in the asymmetric unit. These symmetry reductions provided no meaningful improvement in merging statistics for the X-ray intensity data, nor was there appreciable reduction in disorder in electron density maps for any of the lower symmetry models considered, so refinement continued in *P*4_2_2_1_2 with two alternate conformations for the entire molecule.

### Molecular dynamics simulations

Molecular dynamic simulations were performed with GROMACS v.2023 (27) within the NMRbox resource (28) using the AMBER forcefield with χ_OL3_ modifications (29). RNA was solvated in a cubic box of TIP3P water molecules, with a minimum distance of 1 nm from the RNA to the box edges. K^+^ and Na^+^ ions were added to neutralize the system to achieve a final concentration of 140 and 10 mM respectively. Energy minimization was performed over 50 000 steps using the steepest descent algorithm. Next, 100 ps of NVT (number of particles, volume and temperature) equilibration was applied over 50 000 steps using 2 fs timesteps and a modified Berendsen thermostat that gradually increased temperature from 0 to 300 K. This was followed by 100 ps of NPT (number of particles, volume and temperature) equilibration using 50 000 steps and 2 fs timesteps with a modified Berendsen barostat with pressure maintained at 1 bar. Long-range electrostatic interactions were calculated using the Particle Mesh Ewald method. A production run of 3000 ns was performed with 2 fs timesteps, and trajectory coordinates were saved every 10 ps. Trajectory analysis was carried out using the GROMACS, MDanalysis (30) and Barnaba (31) software packages and visualized with PyMOL v2.4.0 Schrödinger, LLC.

## Results

### Self-assembly of pUG RNA into condensates *in vitro*

**Figure 1.**
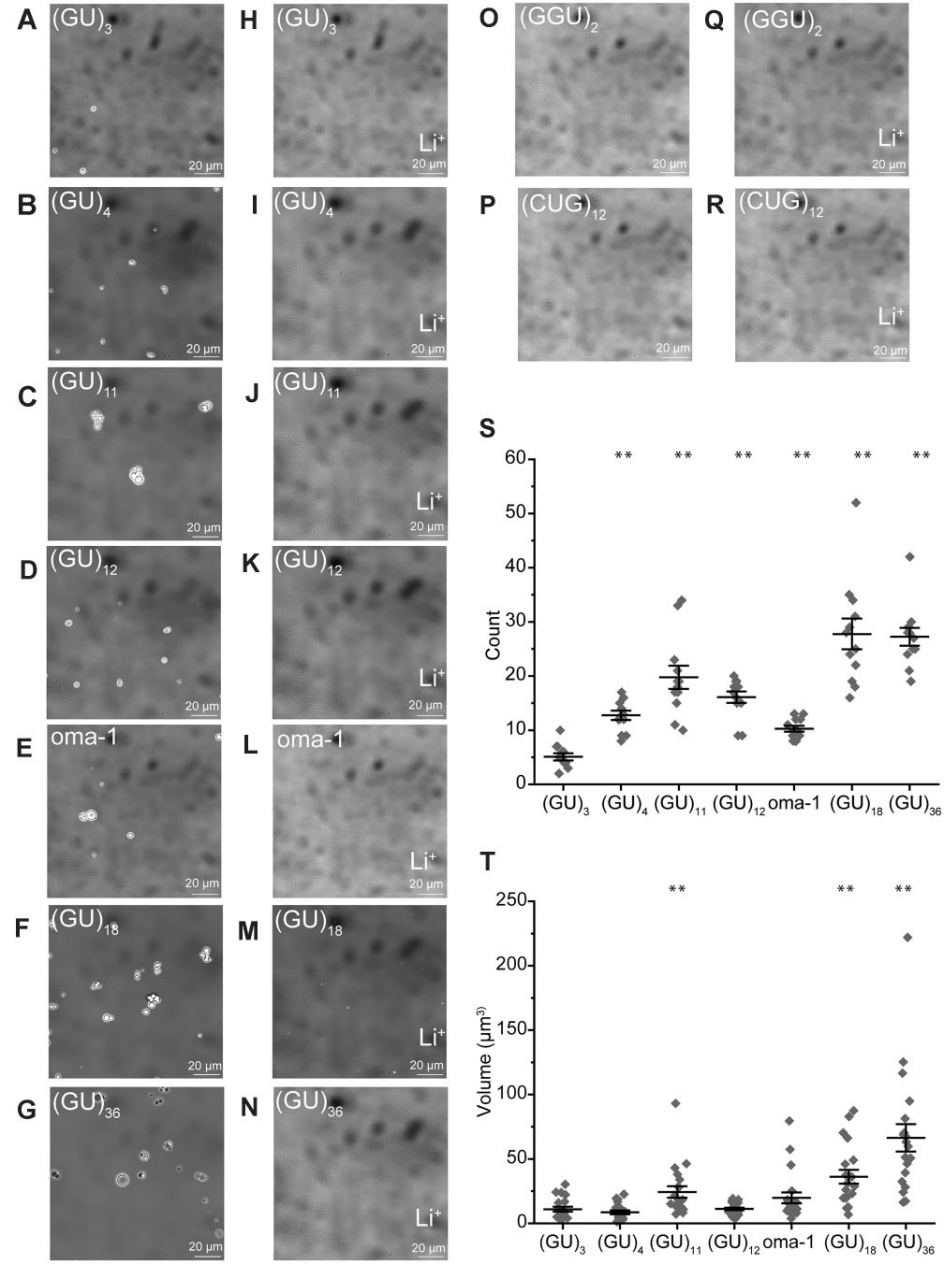
Poly(UG) RNAs form condensates *in vitro*. (**A**–**G**, **O** and **P**) Brightfield differential interference contrast micrographs of (GU)_3_, (GU)_4_, (GU)_11_, oma-1, (GU)_12_, (GU)_18_, and (GU)_36_, (GGU)_2_ and (CUG)_12_ RNAs in K^+^ buffer. (**H**–**N**, **Q** and **R**) Same as (A–G, O and P) except in Li^+^ buffer. Scale bars, 20 μm. Invariant dark spots observed in the background of all images are due to the internal optics of the microscope. (**S**) Particle count for the different pUG RNAs in K^+^ buffer. (**T**) Particle volume for the different pUG RNAs in K^+^ buffer. Error bars represent mean ± interquartile. A double asterisk (**) indicates a *P*-value less than 0.01, in comparison to (GU)_3_.

We investigated whether pUG RNAs can form condensates *in vitro* under conditions that mimic molecular crowding (150 mM KCl and 10% PEG-8000) (32) using brightfield microscopy. RNA concentrations were 0.23 mg/ml, which corresponds to 120 μM for (GU)_3_ and 10 μM for (GU)_36_ and normalizes for the number of nucleotides. We tested pUG lengths ranging from 3 to 36 repeats and observe K^+^ dependent spherical condensates for all RNAs (Figure 1A–G). Condensation is abolished in Li^+^, consistent with a requirement for G4 formation (Figure 1H–N). To more closely mimic the biological context of a 3′ pUG tail (7,10,11), we tested a 51 nt RNA which has the pUG sequence (GU)_12.5_ attached to the 3′ end of an RNA fragment derived from the oma-1 mRNA (Supplementary Table S1), previously shown to be a target of silencing *in vivo* (11). We have previously shown that the pUG tail region of this oma-1 fragment adopts a pUG fold *in vitro* (33). The oma-1 construct (oma-1) also forms K^+^ dependent condensates (Figure 1E and L), indicating the presence of the additional 5′ sequence does not interfere with pUG condensation.

The hexamer (GGU)_2_ does not form condensates (Figure 1O and Q), despite having a higher guanosine content than the pUG hexamer (GU)_3_ (Figure 1A and H). Therefore, condensation of short pUG RNAs is sequence-dependent and cannot be simply attributed to a high guanosine content. We also tested the control sequence (CUG)_12_, which does not form condensates as previously described (Figure 1P and R) (13). Collectively, these data show that short pUG sequences have a unique ability to efficiently form G4 condensates. We quantified both the number and size of condensates as a function of pUG length (Figure 1S and T). The number and size of condensates generally increase with pUG length, with the exception of (GU)_12_ and oma-1, which form smaller condensates than (GU)_11_. This may be due to the fact that twelve guanosines are the minimal number of guanosines required to adopt the highly compact intramolecular pUG fold (11). Interestingly, (GU)_3_, (GU)_4_ and (GU)_11_ are not long enough to adopt the intramolecular pUG fold (11), but still form condensates. We therefore hypothesized that condensation of pUG RNAs may involve some other type of G4 architecture.

**Figure 2.**
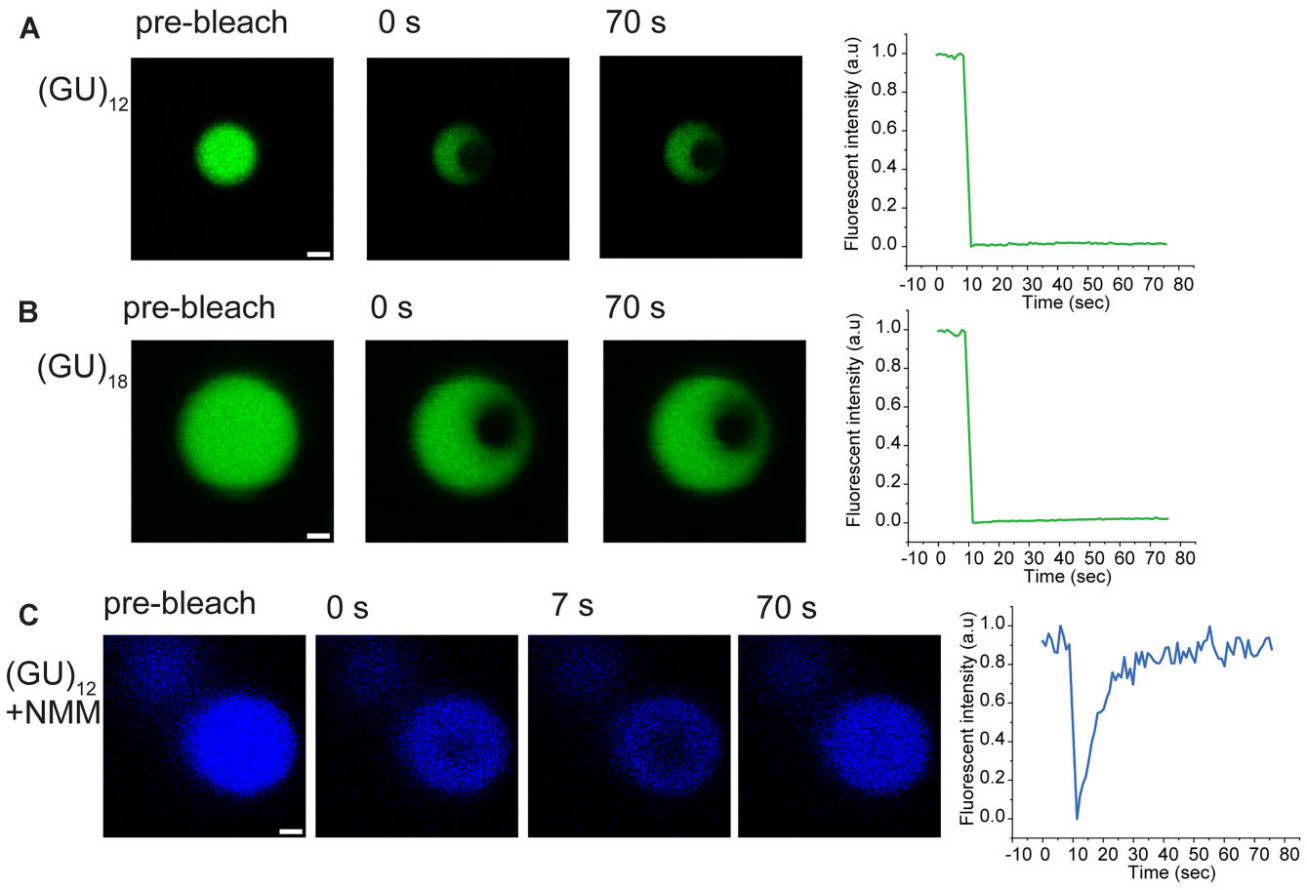
Fluorescence recovery after photobleaching (FRAP) experiments of 5′-fluorescein pUG RNA. (**A**) (GU)_12_, (**B**) (GU)_18_ and (**C**) unlabeled (GU)_12_ with NMM. Fluorescence recovery plots are shown.

FRAP was used to determine the physicochemical state of the (GU)_12_ and (GU)_18_ RNAs, which correspond to pUG tail lengths that are active for gene silencing (11). For these experiments, the pUG RNAs were labeled with 5′ fluorescein dyes. Both RNAs formed condensates and showed no fluorescence recovery over 70 seconds. Therefore, these RNAs cannot freely diffuse and are gel-like solids (Figure 2A and B) (34). To further confirm that G4 structures are present in these condensates, we performed the same experiments with unlabeled (GU)_12_ RNA and incubated the condensates with the G4-specific probe *N*-methyl mesoporphyrin IX (NMM) (35,36). NMM fluorescence increases ∼60-fold when stacked on G4s (37), and binds to the pUG fold (11) and other types of G4s (38,39). Although the NMM fluorescence is not as bright as fluorescein, the FRAP data clearly show the pUG condensates bind NMM, confirming they contain G4 structures (Figure 2C). The rapid recovery of NMM fluorescence shows that the small molecule probe can freely diffuse on and off the RNA without disrupting condensate formation.

### Molecular basis of pUG self-assembly

**Figure 3.**
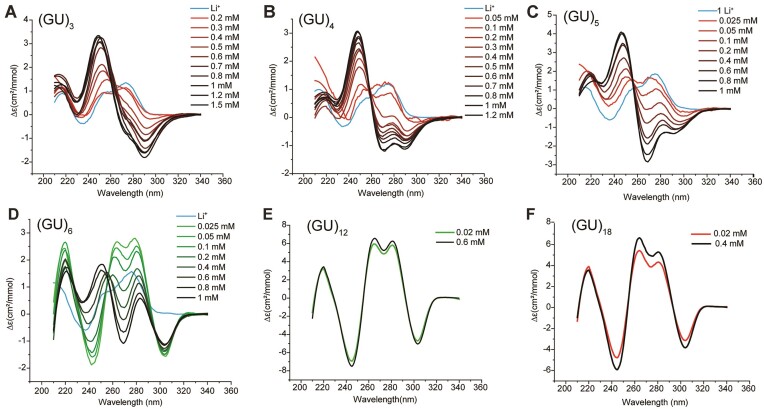
RNA secondary structure as a function of GU repeat length and concentration, measured by CD spectroscopy. All samples were in 140 mM KCl,10 mM NaCl and 2 mM MgCl_2_, except for the Li^+^ samples, which were in 150 mM LiCl. (**A**) (GU)_3_, (**B**) (GU)_4_, (**C**) (GU)_5_, (**D**) (GU)_6_, (**E**) (GU)_12_, (**F**) (GU)_18_.

Since pUGs with 3–11 repeats are too short to adopt the intramolecular pUG fold (11) but still form G4 condensates, we investigated these RNAs for secondary structure by circular dichroism (CD) spectroscopy. The short pUG RNAs (GU)_3_, (GU)_4_, and (GU)_5_ all form concentration-dependent G4 secondary structures and exhibit a major positive peak at 250 nm, typical of right-handed parallel G4s with *anti*-*anti* stacking (Figure 3A–C) (40). In contrast, the (GU)_12_ pUG fold is a left-handed parallel G4 with *syn*-*syn* stacking (11) and displays a prominent negative 250 nm peak in its CD spectrum (Figure 3E). Note for example the CD spectra of (GU)_4_ and (GU)_12_ are nearly perfect mirror-image inversions of each other (compare Figure 3B and E). Like other G4 structures, the right-handed pUG G4s require K^+^ ions and are destabilized in Li^+^ (Figure 3A–C). The right-handed pUG G4s still fold when attached to additional 5′ nucleotides, as demonstrated by comparison of the K^+^ dependent folding of (GU)_5_ and AA(GU)_5_ by CD (Supplemental Figure S1).

The concentration-dependence of pUG G4 folding for (GU)_3_, (GU)_4_, (GU)_5_ and (GU)_6_ indicates these structures are intermolecular (Figure 3A–D). As pUG length increases from 3–6 repeats, new CD peaks are observed, consistent with formation of new quartets (Figure 3A–D). Surprisingly, two different structures are observed for (GU)_6_. At the lowest concentration measured (25 μM) the CD spectrum displays 5 peaks that precisely correspond to the intramolecular pUG fold of (GU)_12_ (Figure 3D, E). These data indicate that (GU)_6_ can dimerize into an intermolecular version of the left-handed pUG fold. However, as the (GU)_6_ concentration is increased the pUG fold signal diminishes, and the structure converts into a right-handed G4 with a positive peak at 250 nm that is characteristic of the right-handed pUG G4 spectra (Figure 3A–D).

We have previously shown that (GU)_12_ forms an intramolecular pUG fold which is concentration independent, K^+^ dependent, and does not fold in Li^+^ (11). Spectra of the pUG fold at low and high concentrations are shown (Figure 3E). Like (GU)_12_, (GU)_18_ also adopts an intramolecular pUG fold at both low and high concentrations (Figure 3F). In summary, (GU)_3_, (GU)_4_, (GU)_5_ and (GU)_6_ can all form right-handed intermolecular pUG G4 structures, while (GU)_6_ can also adopt a dimeric pUG fold. Both (GU)_12_ and (GU)_18_ form stable intramolecular left-handed pUG folds (11). A slight increase in pUG fold signal is observed for (GU)_18_ at higher concentrations (Figure 3F), which could be due to formation of pUG folds through both intramolecular (GU)_12_ and dimeric (GU)_6_ interactions.

**Figure 4.**
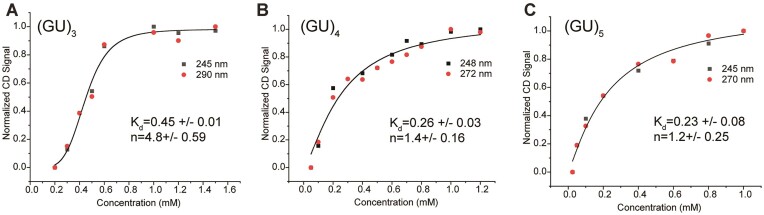
Concentration dependence of pUG-G4 formation, measured by CD spectroscopy. Y axis is the normalized magnitude change of the CD signal. Data are fit to the Hill equation for: (**A**) (GU)_3_, (**B**) (GU)_4_ and (**C**) (GU)_5_.

The apparent equilibrium dissociation constants (*K*_d_) and Hill coefficients (*n*) for two-state folding of (GU)_3_, (GU)_4_ and (GU)_5_ can be fit to the Hill equation (Figure 4A–C). As the number of pUG repeats increases, self-association becomes stronger, and the equilibrium dissociation constants decrease from 450 to 230 μM (Figure 4). The folding of (GU)_3_ is highly cooperative with a Hill coefficient of ∼4.8, consistent with formation of a multi-stranded quadruplex structure via a predominately two-state folding pathway. However, the Hill coefficients for (GU)_4_ and (GU)_5_ are 1.4 and 1.2 (Figure 4B and C). This decreasing trend in cooperativity is consistent with the presence of folding intermediates, as described previously for other G4 structures (41). Therefore, as pUG length increases, so does structural diversity and folding complexity.

### Structure of pUG G4 RNA

**Figure 5.**
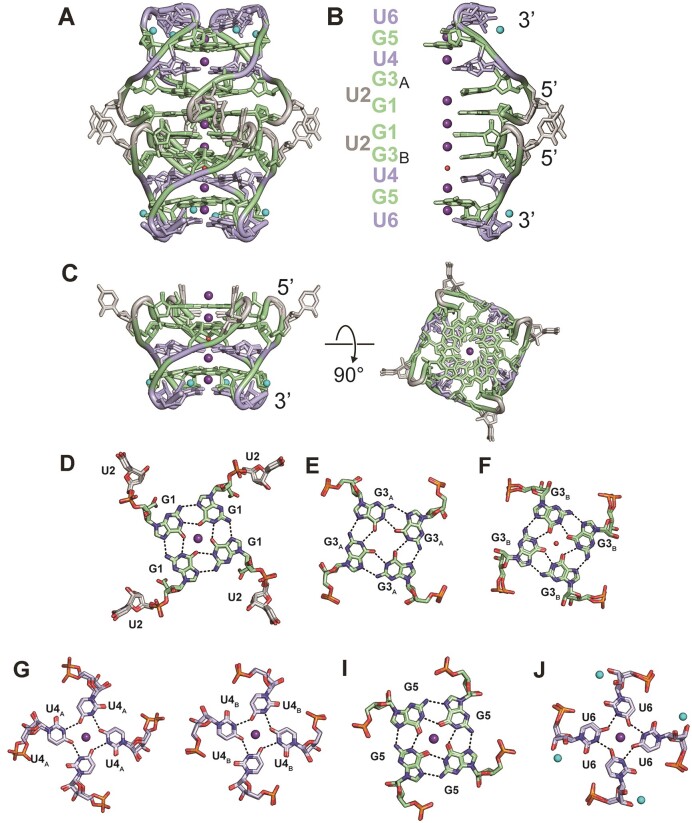
(**A**) Structure of the dimeric intermolecular parallel quadruplex formed by (GU)_3_. G’s are green, U’s in quartets are blue, and bulged Us are light gray. (**B**) Sequence and cut-away view showing the stacking of two strands of (GU)_3_. The top strands are denoted with a subscript A and the bottom strands are denoted with subscript B. (**C**) Structure of (GU)_3_ quadruplex B shown from the side and top-down. (**D**) G1 quartet and bulged U2 (conformation is the same in A and B). (**E**) G3 quartet for subunit A. (**F**) G3 quartet for subunit B with the central water molecule shown in red. (**G**) U4 quartet in subunit A. (**H**) U4 quartet in subunit B. (**I**) G5 quartet hydrogen bonding (conformation is the same in A and B). (**J**) U6 quartet (conformation is the same in A and B). Potassium ions are shown in purple, sodium ions are cyan.

We determined the structure of the (GU)_3_ pUG G4 by single crystal x-ray diffraction methods to a maximum resolution of 1.05 Å (Table 1, Supplemental Figure S2, Figure 5). The structure contains eight hexanucleotide strands (48 nucleotides) and forms a dimer of right-handed parallel quadruplexes (Figure 5). The right-handed intermolecular structure is consistent with the concentration dependent CD data (Figures 3 and 4). The dimer is a 5′–5′ head-to-head stack of G4s mediated through a shared potassium ion coordinated between the first quartets (Figure 5A and B). A total of seven potassium ions are coordinated between the quartets, and eight sodium ions are bound at the 3′ ends (Figure 5A–C). The structure contains six G-quartets, four U-quartets and eight bulged uridines. The right-handed parallel pUG G4 forms a continuous stack of G and U quartets except for the U2 nucleotides which bulge out and stack on each other. Seven distinct conformations are observed for the 10 G and U quartets (Figure 5D–J), as described below.

**Table 1.** Data collection and refinement statistics

	GUGUGU quadruplex PDB 7KSD	Holmium phasing derivative
**Data collection**		
Wavelength (Å)	0.9762	1.3051
Resolution range (Å)^a^	21.7–1.05 (1.09–1.05)	56.7–1.41 (1.43–1.41)
Space group	*P*42_1_2	*P*42_1_2
Unit cell dimensions (Å)	32.8, 32.8, 58.0	32.9, 32.9, 56.7
Total reflections^a^	29 415 (1827)	140 188 (2,282)
Unique reflections^a^	14 726 (937)	6 221 (203)
Multiplicity *y*^a^	12.4 (3.5)	22.5 (11.2)
Completeness (%)^a^	95.75 (68.98)	96.3 (68.8)
Mean *I*/σ(*I*)^a^	17.69 (2.14)	16.4 (1.1)
Wilson *B*-factor	9.55	11.1
*R*-merge^a^	0.01436 (0.2517)	0.13 (2.33)
CC_1/2_^a^	1 (0.881)	0.999 (0.273)
FOM		0.76
		
**Refinement**		
Resolution^a^	21.73–1.05 (1.09–1.05)	
No. reflections^a^	29 415 (1827)	
*R*_work_/*R*_free_^a^	0.15/0.16 (0.20/0.23)	
Total number of atoms^b^	556	
RNA	504	
Ligands	9	
Water	43	
Clashscore	9.57	
RMS(bonds)	0.006	
RMS(angles)	1.14	
Average *B* factor (Å^2^)	13.57	
RNA	12.2	
Ions	9.60	
Solvent	22.79	

^a^Values shown in parentheses are for the highest resolution shell.

^b^Non-hydrogen atoms.

The two stacked pUG G4s are in different conformations, hereafter referred to as A and B (pUG G4_A_ and pUG G4_B_). There are no ions between the G3 and U4 quartets, and only in pUG G4_B_ is there is a single buried water molecule at this location (Figure 5F and Supplemental Figure S3C and D). The water binding site is created by the closer stacking of the G3_B_ and U4_B_ quartets (3.4 Å versus 3.9 Å for G3_A_ and U4_A_) (Supplemental Figure S3E and F). The G3_A_ quartet has C2′-endo sugar puckers while the G3_B_ quartet has C3′-endo sugar puckers (Supplemental Figure S3E and F). The stacking arrangements of the G1 and G3 quartets are also slightly different in pUG G4_A_ and G4_B_ (Supplemental Figure S3A and B). The water binding site has cubically arranged carbonyl oxygens that accept hydrogen bonds from water, whereas the K^+^ binding sites have carbonyl oxygens arranged in octahedral geometries for ion coordination (Supplemental Figure S3A, B and D).

While RNA is understood to be dynamic, it is relatively rare to resolve the result of such motions in a crystal (42). The resolution of the pUG G4 structure is sufficient to reveal slightly different conformations within the individual strands of the structure. These differences map predominately to the G3 and U4 quartets. In pUG G4_A_, the U4 quartet has 2 different but equally populated phosphate positions (Figure 5G, Supplemental Figure S2F and Supplemental Figure S4). In pUG G4_B_, the G3 quartet has 2 different phosphate positions (Figure 5F and Supplemental Figure S2E). We interpret this structural heterogeneity as frozen snapshots of RNA dynamics in the crystal. To further explore and visualize these dynamics, we performed molecular dynamics simulations of the pUG G4 RNA structure for 3 μs (Supplemental Video 1). The dimeric structure is stable over the course of the simulation, including the coordinated potassium ions and buried water molecule, while the partially hydrated sodium ions rapidly diffuse away from the RNA. The most dynamic region of the molecule is the bulged U2 nucleotide, as expected (Supplemental Figure S5A and B). The G3_A_ sugar puckers remain predominately C2′ endo and only rarely sample the C3′ endo conformation (Supplemental Figure S6). On the other hand, the G3_B_ sugar puckers sample the C2′ and C3′ endo conformations almost equally (Supplemental Figure S6). These data suggest the buried water molecule helps to stabilize the G3_B_ C3′ endo conformation. Trapped water molecules can have important structural roles but have only been observed in a small number of nucleic acid structures (43,44).

In summary, the pUG G4 crystal structure is an 8-stranded asymmetric dimer of quadruplexes, with each strand exhibiting two slightly different conformations. Thus, a total of 16 strands were modeled in the pUG G4 structure. The glycosidic torsion angles and sugar puckers of all nucleotides are listed in Table 2. In addition to the seven coordinated potassium ions, eight partially hydrated sodium ions are bound to the ribose groups of the two 3′ terminal U6 quartets (Figure 5J, Supplemental Figure S2I and Supplemental Figure S3G). The 3′ terminal G5 and U6 quartets and sodium binding sites are essentially identical in both pUG G4_A_ and G4_B_.

**Table 2.** Nucleotide conformations in the GUGUGU quadruplex

Nucleotide	Backbone direction (relative to 5′ end)	Glycosidic torsion angle	Sugar pucker
G1		*Syn*	2′-endo
U2	Inverted	*Anti*	2′-endo
G3_A_/G3_B_	Inverted	*Anti*	2′/3′-endo
U4	Inverted	*Anti*	3′-endo
G5	Inverted	*Anti*	3′-endo
U6		*Anti*	2′-endo

## Discussion

We have shown that pUG RNAs can self-associate into G4s and form K^+^-dependent condensates. Self-association of pUG RNA involves weak interactions that may occur in crowded condensates such as *Mutator* foci and may perhaps be stabilized by protein interactions. These weak pUG interactions include formation of multi-stranded G4 structures, intermolecular stacking of guanosine quartets, and intermolecular stacking of unpaired uridines (Figure 5A and B). These properties help explain why short pUG RNAs are particularly efficient at forming condensates (Figure 1) and can do so even when appended to the 3′ of unrelated RNA sequence (Figure 1E). In drosophila germ granules, mRNAs of the same sequence have been observed to self-assemble in a phenomenon termed homotypic clustering (45). The molecular basis for homotypic clustering has not yet been explained but does not appear to involve Watson-Crick interactions (45). Therefore, the self-association of RNA in germ granules may be facilitated by non-Watson crick interactions, be protein mediated, or a combination of both.

**Figure 6.**
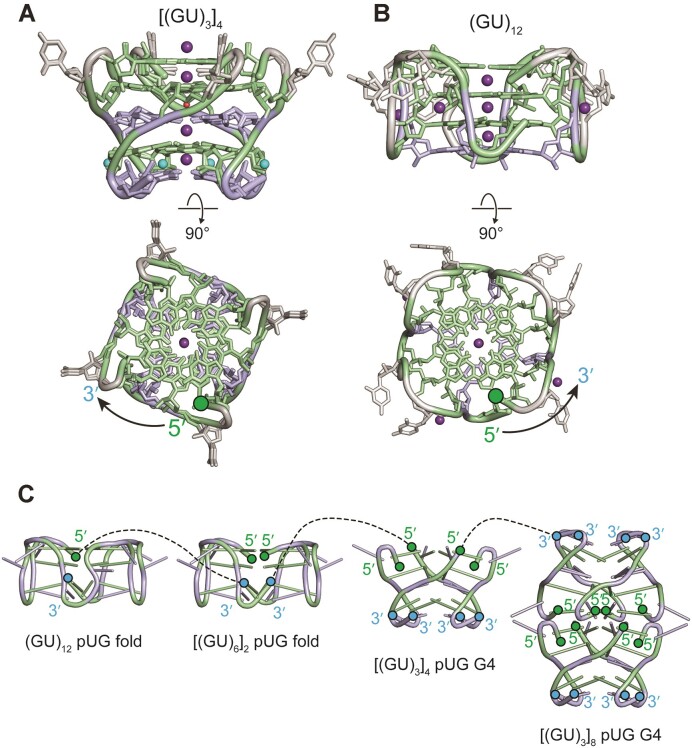
Comparison of the (**A**) pUG G4, and (**B**) pUG fold structures. (**C**) Illustration of some of the possible covalent connections between condensed phase multivalent pUG RNAs shown as dotted lines for the (GU)_12_ pUG fold, dimeric (GU)_6_ pUG fold, [(GU_3_)]_4_ pUG G4 and [(GU_3_)]_8_ pUG G4. 5′ and 3′ ends are marked with green and blue dots.

Comparison of the right-handed intermolecular pUG G4 and left-handed intramolecular pUG fold gives insights into the molecular basis of G4 handedness, as these structures have the same sequence composition but opposite topologies (Figure 6A and B). The pUG fold has a backbone conformation similar to Z-form RNA within the quartets (12). However, the single nucleotide propeller loops between each strand of the pUG fold are right-handed. This mixture of left- and right-handedness maximizes base stacking and hydrogen bonding within the topological constraints imposed by the covalent connections of the single stranded pUG fold. In contrast, the ends of the intermolecular pUG G4 structure have no covalent connections and are topologically relaxed, allowing the structure to extend from three to at least six repeats (Figure 3). Computational studies have suggested that right-handed DNA G4 structures are more stable than left-handed DNA G4s (46), and right-handed topologies are observed for the vast majority of nucleic acid G4 structures in the PDB, with the exception of the pUG fold RNA and a few DNA G4s (46,47). Our observations suggest that topological constraints drive the left-handed segments of the intramolecular pUG fold.

Common features of both the pUG-G4 and pUG fold RNA structures include extensively stacked G and U quartets, backbone inversions, and bulged 5′ uridines (U2) (Table 2, Figure 6A and B). Although the pUG-G4 and pUG fold structures have different handedness, they both have a bulged U2 followed by a backbone inversion at G3 which enables stacking of adjacent G1 and G3 quartets. The bulged U2 conformation is present in both the solution and x-ray structure of the pUG fold so is not driven by crystal contacts (11,33). However, in the (GU)_3_ pUG G4 crystal structure described here, bulged U2 nucleotides from neighboring strands stack (Figure 5A and B), and these uridines in turn make crystal contacts and stack on U2 nucleotides from adjacent molecules. The 5′-5′ stacking interaction between the pUG G4s (Figure 5A and B and Supplemental Figure S3H) has also been previously observed for other G4 RNA structures (48–52). The uridine and guanosine intermolecular stacking interactions likely facilitate pUG condensation (Figures 1 and 2). The backbone inversion near the 3′ end of the pUG G4 structure enables a hydrogen bond to form between the 2′ hydroxyl of the terminal U6 and the 5′ phosphate of the preceding G5 and creates the sodium binding site (Figure 5J). Although this is the first observation of sodium binding to this site, the 3′ inverted uridine quartet appears to be a stabilizing motif and has been previously identified in different G4 structures (48–52).

The formation of intermolecular RNA structure exclusively from GU/UG wobble pairs is not an energetically favorable alternative as the nearest neighbor free energies of adjacent wobble pairs are close to zero (53). On the other hand, G4 structures exhibit extensive base stacking and hydrogen bonding and there is a growing body of evidence for biological roles of G4s in regulating gene expression. Human genes have thousands of long GU repeat sequences (11), and even more short ones. We calculate there are >500 000 examples of the GUGUGU sequence within human genes. In the future, it will be interesting to investigate if pUGs form G4 interactions in human cells.

Our results suggest that pUG G4 interactions may play a role in gene silencing and the organization of *Mutator* foci, where pUG tails can be over 100 nucleotides long (7,10). We hypothesize that the pUG fold and pUG G4 interactions may occur within the same RNA, or between multiple RNAs (Figure 6C). These multivalent interactions may aid in the formation of condensates and could provide an explanation for the increased silencing efficiency of longer pUG tails. Such intermolecular pUG interactions might be resolved by the helicases that reside within germ granules and are required for RNAi (54). Finally, given the abundance of GU repeat sequences in genomes, it seems likely that pUG G4 interactions may have biological functions that are yet to be identified.

## Supplementary Material

gkae870_Supplemental_Files

## Data Availability

The coordinates and structure factors for the pUG G4 structure have been deposited in the Protein Data Bank and are available under accession code 8VJT. Microscopy data will be shared on reasonable request to the corresponding author.
