# Supplementary material for: Self-assembly and condensation of intermolecular poly(UG) RNA quadruplexes
Source: Nucleic Acids Res. 2024 Oct 7;52(20):12582–91. doi: 10.1093/nar/gkae870 (PMC11551768; doi:10.1093/nar/gkae870)
Supplement: gkae870_Supplemental_Files [file gkae870_supplemental_files.zip › Supplementary_data.pdf]

Self-assembly and condensation of intermolecular poly(UG) RNA quadruplexes

Saeed Roschdi, Eric J. Montemayor, Rahul Vivek, Craig A. Bingman and Samuel E. Butcher

Department of Biochemistry, University of Wisconsin-Madison, Madison, WI 53706 USA

Supplementary Table 1
Supplementary Data Figures 1-6

Supplementary Table 1: RNA oligonucleotide sequences.

RNA	Nucleotide Sequence
**(GU)$_3$**	5′-GUGUGU-3′
**(GU)$_4$**	5′-GUGUGUGU-3′
**(GU)$_5$**	5′-GUGUGUGUGU-3′
**AA(GU)$_5$**	5′-AAGUGUGUGUGU-3′
**(GU)$_6$**	5′-GUGUGUGUGUGU-3′
**(GU)$_{11}$**	5′-GUGUGUGUGUGUGUGUGUGUGU-3′
**(GU)$_{12}$**	5′-GUGUGUGUGUGUGUGUGUGUGUGU-3′
**oma-1**	5′-GGAAACGGUGCCUUUUCAUUCAUCC<br>CGUGUGUGUGUGUGUGUGUGUGUGUG-3′
**(GU)$_{18}$**	5′-GUGUGUGUGUGUGUGUG<br>UGUGUGUGUGUGUGUGUGU-3′
**(GU)$_{36}$**	5′-GUGUGUGUGUGUGUGUGUGUGUGUGUG<br>UGUGUGUGUGUGUGUGUGUGUGUGUGU<br>GUGUGUGUGUGUGUGUGU-3′

Supplemental Figure 1

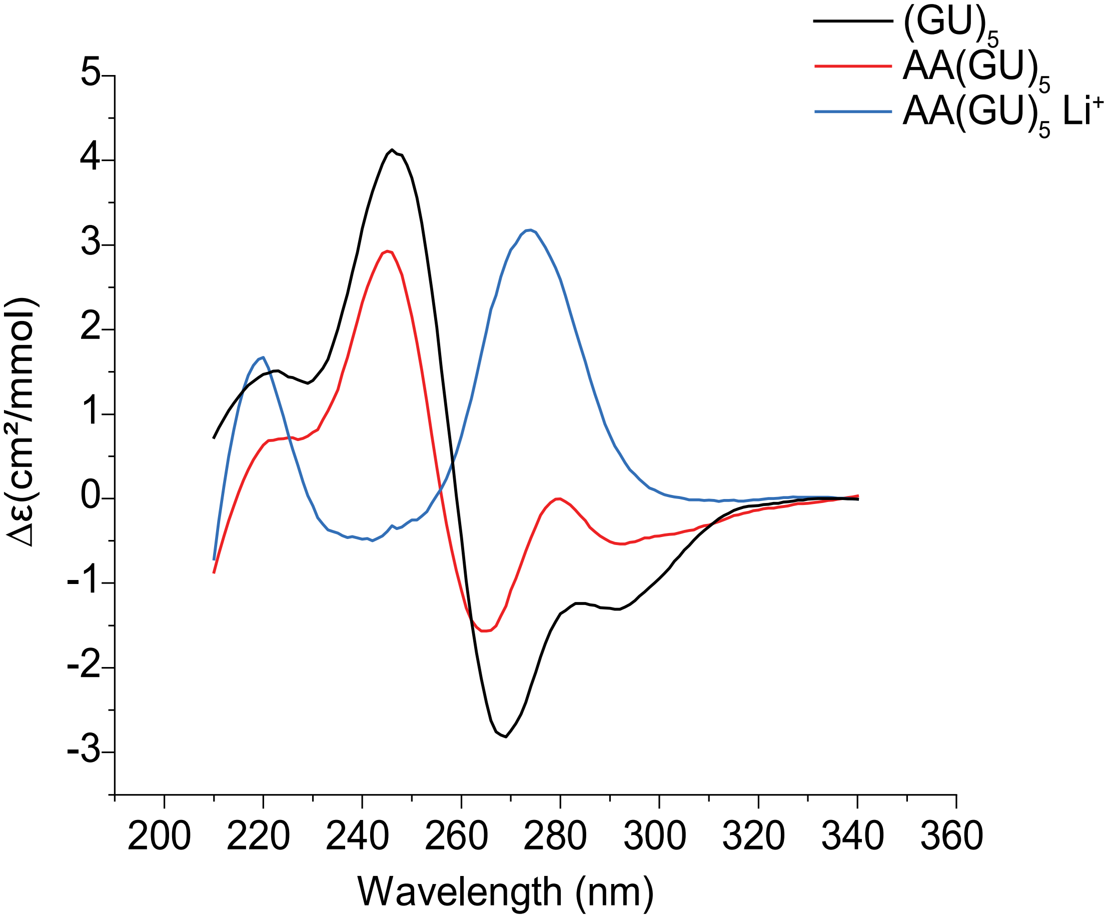

Supplementary Figure 1. RNA secondary structure of $(GU)_5$ compared to $(GU)_5$ with a 5' AA addition, measured by CD spectroscopy. All samples were in 140 mM KCl,10 mM NaCl, 2 mM $MgCl_2$, and 20 mM bis-tris pH 7.0, except for the Li^+ samples, which were in 150 mM LiCl and 20 mM bis-tris pH 7.0.

Supplemental Figure 2

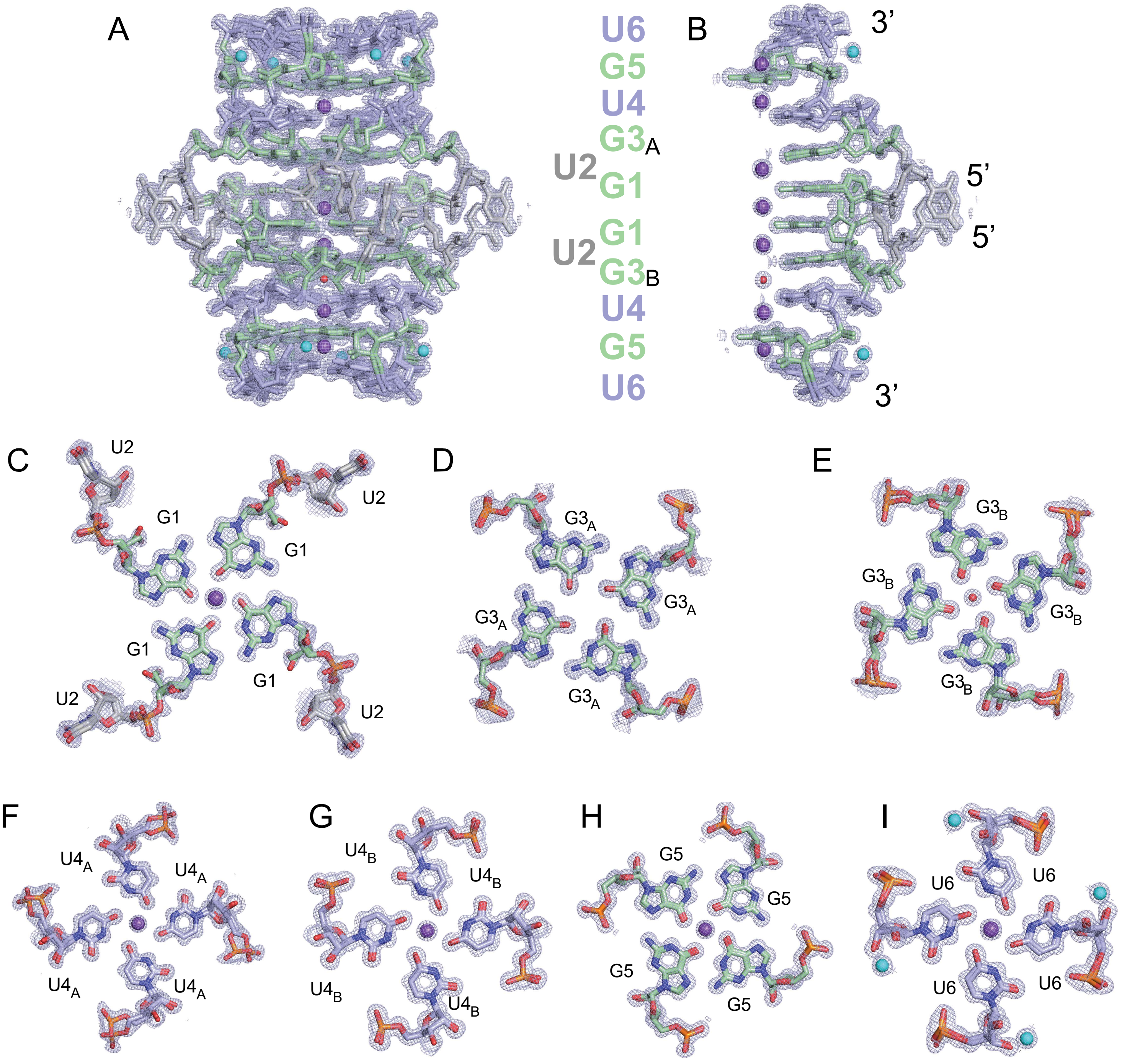

Supplemental Figure 2. 1.05 Å 2Fo-Fc electron density map contoured to 1.6 Å. Views are same as in Figure 5.

Supplemental Figure 3

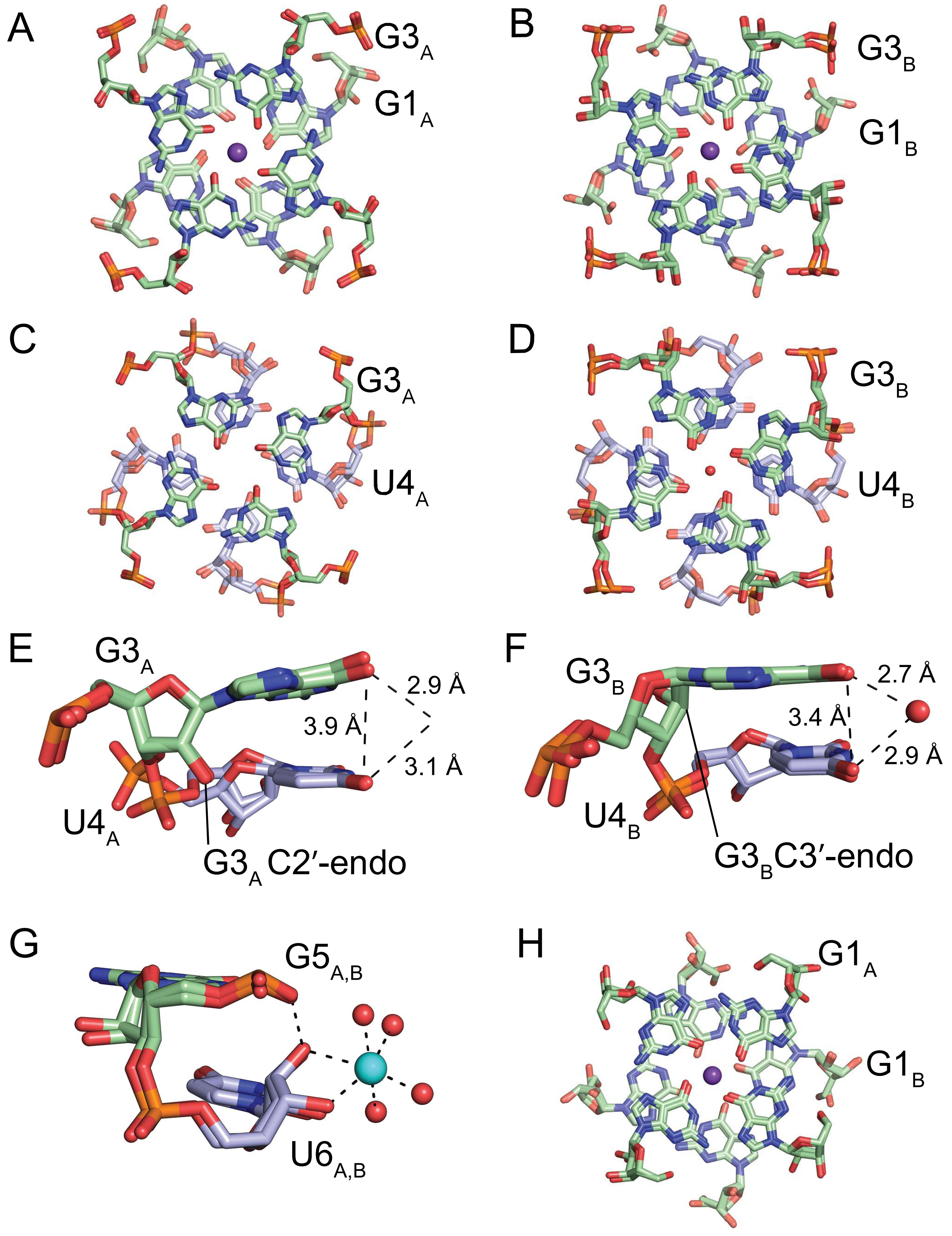

Supplemental Figure 3. (**A**) Stacking of G1A and G3A quartets. (**B**) Stacking of G1B and G3B quartets. (**C**) Stacking of G3A and U4A quartets. (**D**) Stacking of G3B and U4B quartets. (**E**) Distances between G3A and U4A. (**F**) Distances between G3B and U4B. (**G**) Sodium binding site of U6. (**H**) Stacking of G1A and G1B quartets.

Supplemental Figure 4

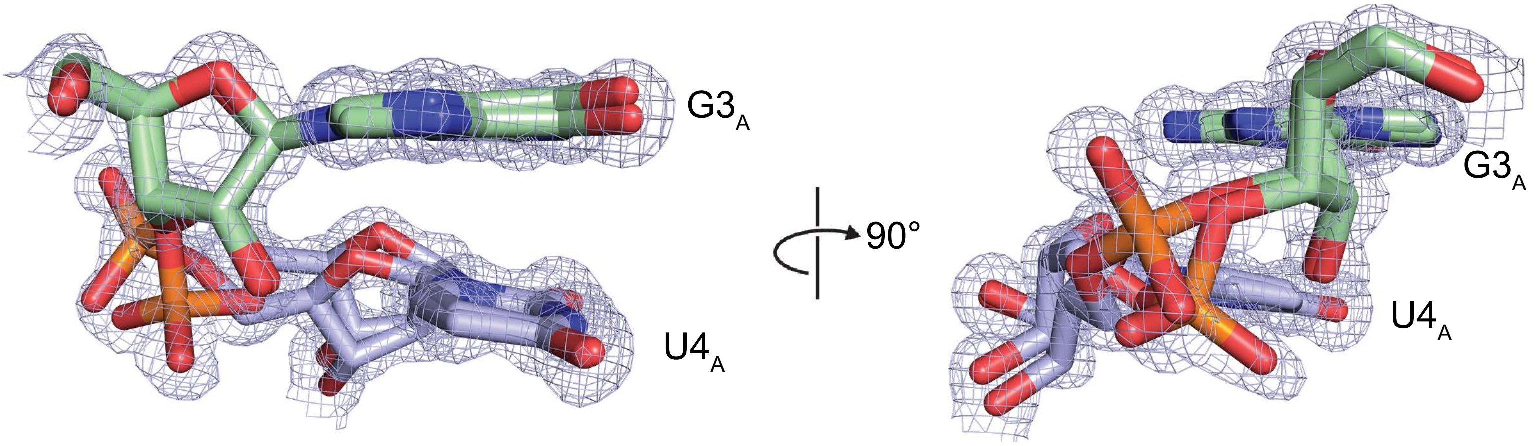

Supplemental Figure 4. Multiple phosphate conformations observed for $U4_A$. 1.05 Å 2Fo-Fc electron density map contoured to 1.6 Å.

Supplemental Figure 5

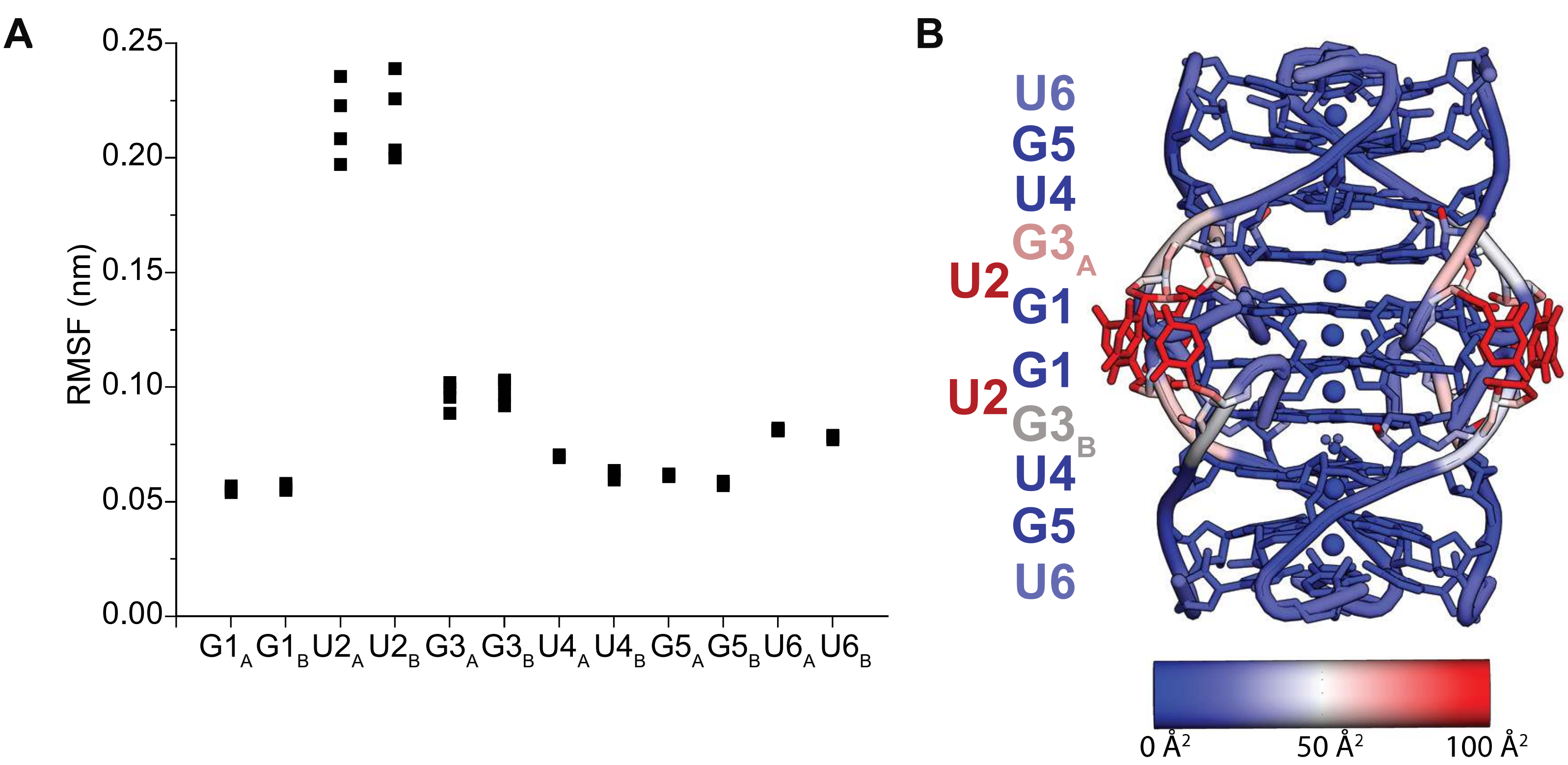

Supplementary Figure 5: Analysis of pUG-G4 structure using molecular dynamic simulations. (**A**) Root mean square fluctuation (RMSF) of individual residues within the different strands of pUG-G4A and pUG-G4B. (**B**) B-factors of individual atoms, shown in blue-white-red gradient from lowest to highest.

Supplemental Figure 6

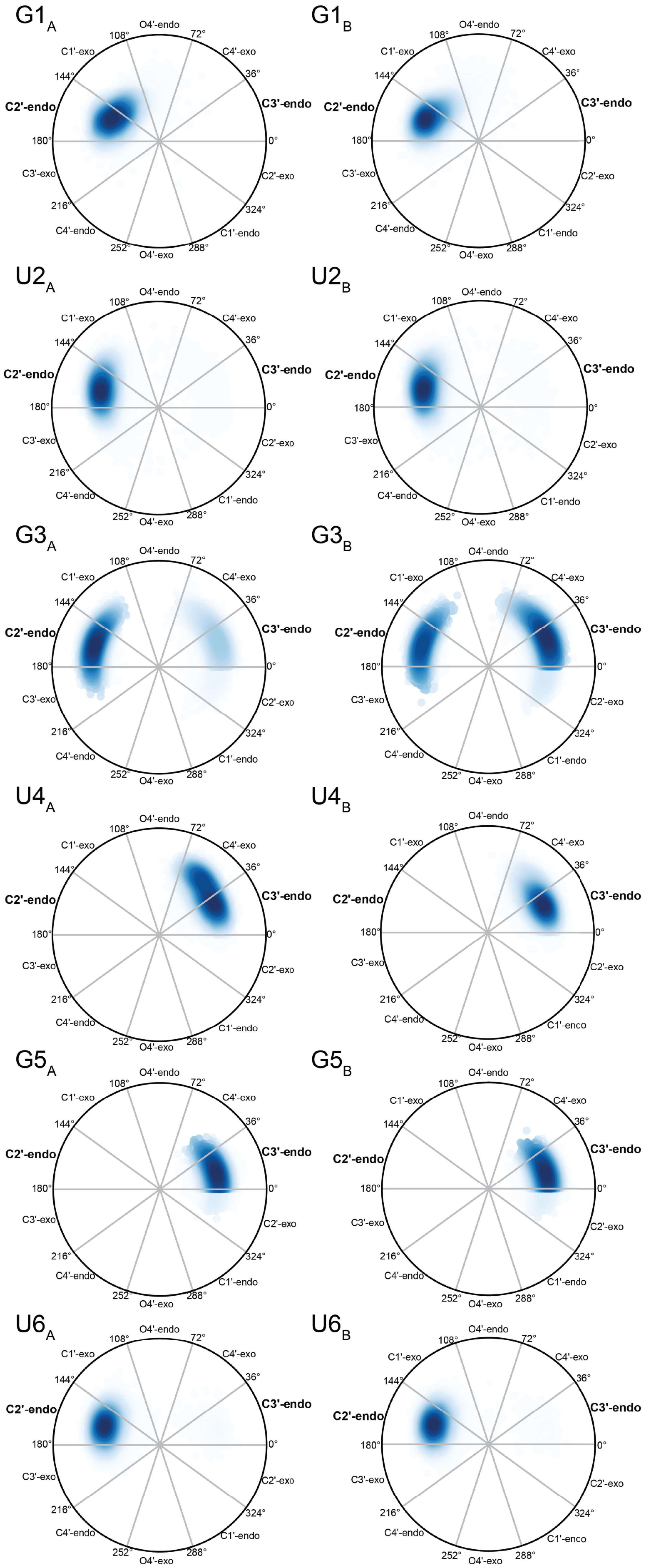

Supplementary Figure 6: Circular histograms of sugar pucker pseudorotation angles observed at 1 ns intervals during the 3 microsecond MD simulation, normalized to represent a probability density function.
